# Probiotic *Lactobacillus plantarum* Promotes Intestinal Barrier Function by Strengthening the Epithelium and Modulating Gut Microbiota

**DOI:** 10.3389/fmicb.2018.01953

**Published:** 2018-08-24

**Authors:** Jing Wang, Haifeng Ji, Sixin Wang, Hui Liu, Wei Zhang, Dongyan Zhang, Yamin Wang

**Affiliations:** Institute of Animal Husbandry and Veterinary Medicine, Beijing Academy of Agriculture and Forestry Sciences, Beijing, China

**Keywords:** *Lactobacillus plantarum*, permeability, tight junction, immune response, host defense, microbiota

## Abstract

Weaning disturbs the intestinal barrier function and increases the risk of infection in piglets. Probiotics exert beneficial health effects, mainly by reinforcing the intestinal epithelium and modulating the gut microbiota. However, the mechanisms of action, and especially, the specific regulatory effects of modulated microbiota by probiotics on the intestinal epithelium have not yet been elucidated. The present study aimed to decipher the protective effects of the probiotic *Lactobacillus plantarum* strain ZLP001 on the intestinal epithelium and microbiota as well as the effects of modulated microbiota on epithelial function. Paracellular permeability was measured with fluorescein isothiocyanate-dextran (FD-4). Gene and protein expression levels of tight junction (TJ) proteins, proinflammatory cytokines, and host defense peptides were determined by RT-qPCR, ELISA, and western blot analysis. Short-chain fatty acid (SCFA) concentrations were measured by ion chromatography. Fecal microbiota composition was assessed by high-throughput sequencing. The results showed that pretreatment with 10^8^ colony forming units (CFU) mL^−1^ of *L. plantarum* ZLP001 significantly counteracted the increase in gut permeability to FD-4 induced by 10^6^ CFU mL^−1^ enterotoxigenic *Escherichia coli* (ETEC). In addition, *L. plantarum* ZLP001 pretreatment alleviated the reduction in TJ proteins (claudin-1, occludin, and ZO-1) and downregulated proinflammatory cytokines IL-6 and IL-8, and TNFα expression and secretion caused by ETEC. *L. plantarum* ZLP001 also significantly increased the expression of the host defense peptides *pBD2* and *PG1-5* and pBD2 secretion relative to the control. Furthermore, *L. plantarum* ZLP001 treatment affected piglet fecal microbiota. The abundance of butyrate-producing bacteria *Anaerotruncus* and *Faecalibacterium* was significantly increased in *L. plantarum* ZLP001-treated piglets, and showed a positive correlation with fecal butyric and acetic acid concentrations. In addition, the cell density of *Clostridium sensu stricto* 1, which may cause epithelial inflammation, was decreased after *L. plantarum* ZLP001 administration, while the beneficial *Lactobacillus* was significantly increased. Our findings suggest that *L. plantarum* ZLP001 fortifies the intestinal barrier by strengthening epithelial defense functions and modulating gut microbiota.

## Introduction

The sudden changes in diet and the physical and social environment associated with weaning are significant piglet stressors. Elevated plasma cortisol and corticotropin-releasing factor levels are indices of weaning stress (Van der Meulen et al., 2010). The feed intake of most piglets after weaning is relatively low because of the dietary change from liquid milk to solid feed. Decreased feed and water intake cause small intestinal villous atrophy (Lallès et al., 2004), which in turn results in diminished digestive and absorptive capacities and reduced growth rate. Maternal separation and changes in environment are social and environmental stresses that cause tension in piglets and weaken their immune system. Furthermore, the dietary and environmental changes associated with weaning are associated with a substantial modification of the intestinal microbiota and may cause post-weaning diarrhea and enteric infection (Lallès et al., 2007). Perturbations of the intestinal epithelium, weakened immune system, and modified intestinal microbiota induced by weaning stress can profoundly impact piglet health and growth performance and may, in some cases, lead to mortality (Campbell et al., 2013).

The intestine plays a critical role in the defense against harmful external factors. Poor intestinal defense renders piglets more susceptible to weaning stress, leading to infection and disease. In post-weaning piglets, transepithelial electrical resistance (TER) significantly decreases while paracellular permeability increases (Hu et al., 2013). The expression of the tight junction (TJ) proteins occludin, claudin-1, and ZO-1 decreases during weaning, and as a result, barrier integrity is impaired. This facilitates pathogen penetration and permits bacteriotoxins to enter the body. Weaned piglets also exhibit elevated expression of the proinflammatory cytokines TNF-α and IL-6 (Hu et al., 2013), which is associated with weak epithelium and inflammatory disease. Furthermore, weaning lowers intestinal microbial diversity and alters the microbiota composition in that the abundance of obligate anaerobic bacteria decreases and that of facultative anaerobic bacteria increases (Winter et al., 2013), which weakens intestinal function. For instance, *Lactobacillus* spp. and other beneficial bacteria play an important role in protecting against intestinal pathogens, and the reduction in their abundance after weaning enhances disease risk (Konstantinov et al., 2006). *S. enterica* and *E. coli* are two major pathogens infecting piglets. An increased abundance of these pathogens in the intestine often results in severe infection.

Evidence indicates that the consumption of probiotic bacteria contributes to intestinal function by maintaining paracellular permeability, enhancing the physical mucous layer, stimulating the immune system, and modulating resident microbiota composition and activity (Boirivant and Strober, 2007). The regulatory effects of probiotics on human, pig, and chicken intestinal homeostasis have been studied extensively (Van Baarlen et al., 2013; Cisek and Binek, 2014; Gresse et al., 2017), and interactions between probiotic and commensal bacteria and the epithelial barrier are thought to be the main underlying mechanism. *Lactobacillus* is a predominant indigenous bacterial genus found in the human and animal gastrointestinal tract, and species of this genus are commonly used as probiotics. *In vitro* and *in* *vivo* studies in various cell lines and animal models have demonstrated that *L. plantarum* MB452, *L. casei*, *L. rhamnosus* GG, and *L. reuteri* I5007 affect TER and epithelial permeability and modulate TJ protein expression and distribution (Anderson et al., 2010; Eun et al., 2011; Patel et al., 2012; Yang et al., 2015). *Lactobacillus spp.* boost the immune system by promoting the expression of anti-inflammatory cytokines, such as IL-10 and IFN-γ (*Lactobacillus* GG, Kopp et al., 2008; *L. rhamnosus* CRL1505, Villena et al., 2014), or by inhibiting that of pro-inflammatory cytokines, such as IL-6, IL-8, and TNF-α (*L. reuteri* LR1, Wang et al., 2016; *L. plantarum* 2142, Farkas et al., 2014). *L. reuteri* I5007 and *L. plantarum* DSMZ 12028 can modulate the synthesis of antimicrobials by the intestinal epithelium (Paolillo et al., 2009; Liu et al., 2017). Multiple studies have confirmed that *Lactobacillus* spp., such as *L. salivarius* UCC118 and *L. acidophilus*, significantly modulate resident intestinal microbiota composition and activity (Riboulet-Bisson et al., 2012; Li et al., 2017). Thus, through enhancing intestinal epithelial function, probiotics improve host health. However, the effects of probiotics on intestinal barrier function are strain-dependent and not ubiquitous. The unique effects of specific strains on the intestinal epithelium and microbiota, and whether the modulated microbiota affect intestinal epithelial function remain unclear.

The results of our previous studies indicated that dietary supplementation with *Lactobacillus plantarum* ZLP001 isolated from healthy piglet intestinal tract (Wang et al., 2011) improves growth performance and antioxidant status in post-weaning piglets (Wang et al., 2012). However, its impact on intestinal barrier function and microbiota, and the interaction between barrier function and microbiota after *L. plantarum* ZLP001 treatment remained to be investigated. In this study, the impact of *L. plantarum* ZLP001 on intestinal epithelial function was evaluated by measuring gut permeability and the expression of TJ proteins, inflammatory cytokines, and host defense peptides (HDP). Further, we evaluated the ability of this strain to regulate microbiota composition and community structure. The regulatory effects of the microbiota modulated by the probiotic strain on intestinal epithelium function were also analyzed.

## Materials and Methods

### Bacteria and Culture Conditions

*L. plantarum* ZLP001 was isolated in our laboratory from the intestine of a healthy piglet. It was identified by the China Center of Industrial Culture Collection (Beijing, China) and preserved in the China General Microbiological Culture Collection Center (CGMCC No. 7370). It was grown in improved De Man, Rogosa, and Sharpe liquid medium (10 g peptone, 5 g yeast powder, 20 g glucose, 10 g beef extract, 5 g sodium acetate, 2 g ammonium citrate dibasic, 2 g dipotassium phosphate, 0.58 g magnesium sulfate, 0.19 g manganese sulfate, 1 mL of Tween 80, and water to 1,000 mL; pH 6.5) at 37°C under anaerobic conditions.

Enteropathic *E. coli* strain expressing F4 (F4+ ETEC), serotype O149:K91, K88ac, was obtained from the China Veterinary Culture Collection Center. It was grown in Luria-Bertani medium (Oxoid, Basingstoke, United Kingdom) at 37°C.

### Cell Line and Culture Conditions

The porcine intestinal epithelial cell line (IPEC-J2) used in this study was purchased from JENNIO Biological Technology (Guangzhou, China). It was originally derived from the jejuna of neonatal piglets. The cells were cultured in DMEM/F12 (Dulbecco’s modified Eagle’s medium/nutrient mixture F-12, a 1:1 mixture of DMEM and Ham’s F-12; Invitrogen, Carlsbad, CA, United States) supplemented with 10% fetal bovine serum (FBS; Invitrogen, Carlsbad, CA, United States), streptomycin (100 μg mL^−1^), and amphotericin B (0.5 μg mL^−1^). IPEC-J2 cells were cultured at 37°C in a 5% CO_2_/95% air atmosphere and 90% relative humidity. Cells were separated at each passage with 0.25% w/v trypsin (Invitrogen, Carlsbad, CA, United States) and replenished with fresh media every 2–3 days.

### Paracellular Permeability Determination

Changes in paracellular permeability after *L. plantarum* ZLP001 treatment were determined with fluorescein isothiocyanate-dextrans (FD-4; average molecular mass, 4.4 kDa; Sigma-Aldrich Corp., St. Louis, MO, United States) according to the method reported by Wang et al. (2016) with some modifications. IPEC-2 cells were seeded into 6-well Transwell insert chambers (0.4 μm pore size; Corning, Inc., Corning, NY, United States) at a density of 2.5 × 10^5^ cells per well and were cultured to form differentiated monolayers. The cells were pretreated or not with *L. plantarum* ZLP001 (LP, 10^8^ CFU mL^−1^) for 6 h and then challenged or not with 10^6^ CFU mL^−1^ ETEC for 3 h. FD-4 was added to the apical sides of the IPEC-J2 cell monolayers at a final concentration of 1 mg mL^−1^. After incubation, medium (100 μL) was sampled from the basolateral chambers, and the FD-4 concentration was quantified using a fluorescence microplate reader (FLx800; BioTek, Winooski, VT, United States). Calibration curves were plotted with an FD-4 gradient series. All experiments were carried out in triplicate.

### IPEC-J2 Cells Treatment With *L. plantarum* ZLP001 and ETEC

IPEC-J2 cells were seeded into 6-well plates (Corning, Inc., Corning, NY, United States) at a density of 2.5 × 10^5^ cells per well. At 80% confluence, the cells were pretreated or not with *L. plantarum* ZLP001 (LP, 10^8^ CFU mL^−1^) at 37°C for 6 h. Then, the cells were washed three times with PBS and the supernatant was removed. The cells were challenged or not with 10^6^ CFU mL^−1^ ETEC at 37°C for 3 h. Fresh medium containing bacteria was prepared by resuspending and diluting collected bacteria in DMEM/F12 without FBS or streptomycin/penicillin. After incubation, the cells were rinsed with PBS three times and collected for subsequent assays. Culture medium supernatants were collected simultaneously. All experiments were carried out in triplicate.

### Determination of mRNA Expression

The mRNA expression levels of TJ proteins, cytokines, and HDPs were determined by quantitative real-time PCR (RT-qPCR). IPEC-J2 cells collected after incubation were lysed with RNAzol (MRC, Cincinnati, OH, United States). Total RNA was extracted following the manufacturer’s instructions. RNA concentrations were determined with a NanoDrop spectrophotometer (Thermo Fisher Scientific, Waltham, MA, United States) and purity was verified by A260:A280 and A260:A230 absorbance ratios. The RNA was reverse transcribed with an iScript cDNA Synthesis Kit (Bio-Rad Laboratories Ltd., Hercules, CA, United States) according to the manufacturer’s instructions. qPCR was performed using iTaq Universal SYBR Green Supermix (Bio-Rad Laboratories Ltd., Hercules, CA, United States) on a QuantStudio 3 real-time PCR system (Thermo Fisher Scientific, Waltham, MA, United States). Porcine-specific primers are listed in **Supplementary Table S1**. The expression of each gene was normalized to that of glyceraldehyde-3-phosphate dehydrogenase (*GAPDH*) to yield a relative transcript level. PCR conditions were 95°C for 10 min followed by 40 amplification cycles (95°C for 30 s, 60°C for 30 s, and 72°C for 20 s). Relative gene expression was calculated by the 2^−ΔΔC_T_^ method.

### Protein Extraction and Immunoblotting

Total protein from IPEC-J2 cells was extracted after the various treatments using a lysis buffer containing 150 mM NaCl, 1% Triton X-100, 0.5% sodium deoxycholate, 0.1% SDS, and 50 mM Tris–HCl adjusted to pH 7.4 and supplemented with a protease inhibitor cocktail (Applygene, Beijing, China). The IPEC-J2 cells were collected into precooled lysis buffer and kept on ice for 30 min. The lysed samples were centrifuged at 4°C and 12,000 × *g* for 5 min to collect the supernatants. Protein concentrations were determined with a Bicinchoninic Acid Protein Assay Kit (Thermo Fisher Scientific, Waltham, MA, United States). After separation on 10% SDS polyacrylamide gel, proteins were electrophoretically transferred to polyvinylidene difluoride membranes (EMD Millipore, Billerica, MA, United States). The membranes were blocked with 5% skim milk and then incubated with primary antibodies overnight (∼12–16 h) at 4°C. They were then incubated with horseradish peroxidase-conjugated secondary antibodies for 1 h at 20–25°C. The antibodies used are listed in **Supplementary Table S2**. Immunoreactive proteins were detected on a ChemiDoc XRS imaging system (Bio-Rad Laboratories Ltd., Hercules, CA, United States) using Western Blotting Luminol Reagent (Santa Cruz Biotechnology, Dallas, TX, United States). Band densities were analyzed with ImageJ (National Institutes of Health, Bethesda, MD, United States). Results were calculated and recorded as the protein abundance relative to β-actin.

### Proinflammatory Cytokine and Porcine β-Defensin 2 (pBD2) Measurement

Proinflammatory cytokines and porcine β-defensin 2 were measured by an enzyme linked immunosorbent assay (ELISA). Cell culture medium supernatant (500 μL) was centrifuged at 4,000 × *g* for 10 min and then passed through a 0.25-μm pore diameter filter (Corning Inc., Corning, NY, United States). The concentrations of interleukin 6 (IL-6), interleukin 8 (IL-8), tumor necrosis factor α (TNF-α), and pBD2 were determined with porcine-specific ELISA Kits (Abcam, Cambridge, United Kingdom), according to the manufacturer’s instructions.

### Animal Groups and Diets

The experimental protocol was reviewed and approved by the Ethics Committee of the Institute of Animal Husbandry and Veterinary Medicine, Beijing Academy of Agriculture and Forestry Sciences, Beijing, PRC. Humane animal care was practiced throughout the trial.

Ten post-weaning piglets (siblings; Large White × Landrace; 8.54 ± 0.58 kg) were assigned to *L. plantarum* ZLP001 treatment or placebo control groups. Each group consisted of two males and three females. Animals were raised at the Beijing Xiqingminfeng Farm (Beijing, China) in a separate room decontaminated prior to the study and were housed at 25–28°C. Each piglet was kept in an individual 1.28-m^2^ pen with a mesh floor. Each pen contained a feeder and a water nipple. Free access to feed and water was provided throughout the 30-day trial. Piglets received a complete feed specially formulated according to the NRC (2012) and the Feeding Standard of Swine (2004). Detailed information about the diet is shown in **Supplementary Table S3**. The control group received a basal diet supplemented with placebo (2 g kg^−1^ feed). The treatment group was administered the basal diet supplemented with freeze-dried *L. plantarum* ZLP001 (5.0 × 10^9^ CFU g^−1^, 2 g kg^−1^ feed).

### Fecal Sample Collection and Microbiota Analysis

Fresh fecal samples were individually collected from piglet recta at the end of the feeding experiment. The samples were immediately transferred to the laboratory and processed for genomic DNA extraction with an E.Z.N.A. Stool DNA Kit (Omega Bio-Tek, Norcross, GA, United States) according to the manufacturer’s instructions. V3+V4 hypervariable sequences of 16S rDNA were amplified by PCR with TransStart FastPfu DNA Polymerase (TransGen Biotech Ltd., Beijing, China) with barcode-modified universal primers (forward: 338F, 5′-ACTCCTACGGGAGGCAGCA-3′; reverse: 806R, 5′-GGACTACHVGGGTWTCTAAT-3′). Amplified products were separated on 2% agarose gels and extracted and purified with an AxyPrep DNA Gel Extraction Kit (Axygen Biosciences, Union City, CA, United States). Barcoded V3+V4 amplicons were sequenced by the paired-end method with Illumina MiSeq at Shanghai Majorbio Bio-pharm Technology Co. (Shanghai, China). Raw sequences were denoised using Trimmomatic and FLASH software and filtered according to their barcodes and primer sequences with QIIME v. 1.5.0. Chimeras were identified and excluded using the UCHIME algorithm v. 4.2.40. Optimized, high-quality sequences were clustered into operational taxonomic units (OTUs) at 97% sequence identity against a subset of the Silva 16S sequence database (Release 119^1^). Taxon-dependent analysis was carried out using the Ribosomal Database Project (RDP) naive Bayesian classifier, with an 80% bootstrap cutoff. Alpha diversity (Shannon and Simpson indices), abundance (Chao1 and ACE indices), and Good’s coverage and rarefaction were analyzed with mothur v. 1.31.2. Principle coordinates analysis (PCoA) was conducted to visualize differences in fecal community composition. PCoA plots were generated on the basis of Bray–Curtis indices. The linear discriminant analysis effect size (LEfSe) algorithm was used to identify the taxa responsible for the differences between the treatment and control groups. The biomarkers used in the present study had an effect-size threshold of two.

### Determination of Fecal Short-Chain Fatty Acid (SCFA) Concentrations

Fecal SCFA concentrations were determined following modified procedures of Qiu and Jin (2002). Half-gram fecal samples were homogenized in 10 mL of double-distilled water. After centrifugation at 12,000 × *g* for 10 min, the supernatants were removed and filtered through 0.25-μm-pore filters (Corning Inc., Corning, NY, United States). Acetic, propionic, and butyric acids were measured with an ion chromatography system (Dionex Corp., Sunnyvale, CA, United States).

### Statistical Analysis

SPSS v. 22.0 (IBM Corp., Armonk, NY, United States) was used for statistical analysis. The FD-4 concentration and mRNA expression levels were analyzed by one-way ANOVA. Fecal SCFA concentrations were analyzed with unpaired Student’s two-tailed *t*-tests. The results are expressed as the mean ± standard error of the mean (SEM). The significance level was *P* < 0.05. Correlations between fecal SCFA concentration and intestinal-associated microbiota were examined with Spearman’s rank-order correlation test in R v. 3.2.1.

## Results

### Effects of *L. plantarum* ZLP001 on Epithelial Permeability

**FIGURE 1:**
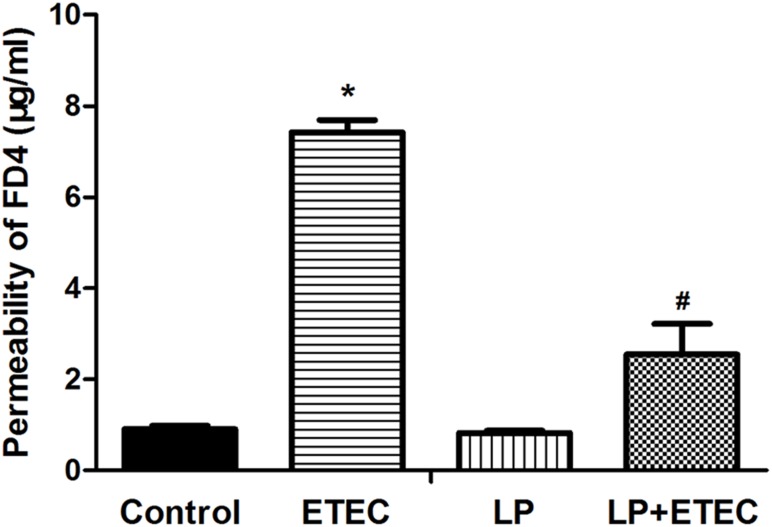
FD-4 diffusion in *L. plantarum* ZLP001-treated IPEC-J2 cells. Cells were left untreated or pretreated with *L. plantarum* ZLP001 (LP, 10^8^ CFU mL^−1^) for 6 h on the apical sides of the cell monolayers and then challenged or not with 10^6^ CFU mL^−1^ ETEC for 3 h. FD-4 concentrations in the basal compartments were measured. Values are shown as the means ± SE of three independent experiments. ^∗^*P* < 0.05 vs. non-treated controls; ^#^*P* < 0.05 vs. ETEC alone.

FD-4 diffusion is a good indicator of paracellular permeability. Therefore, FD-4 transport was measured in this study to evaluate the protective effect of *L. plantarum* ZLP001 on epithelial integrity (**Figure 1**). FD-4 concentrations in the *L. plantarum* ZLP001-treated group were not significantly different from those in the untreated control. When IPEC-J2 cells were exposed to 10^6^ CFU mL^−1^ ETEC alone, FD-4 permeation was significantly increased relative to that of the control group (*P* < 0.05). Pretreatment with *L. plantarum* ZLP001 (10^8^ CFU mL^−1^) significantly counteracted this permeation.

### Effects of *L. plantarum* ZLP001 on TJ Expression

**FIGURE 2:**
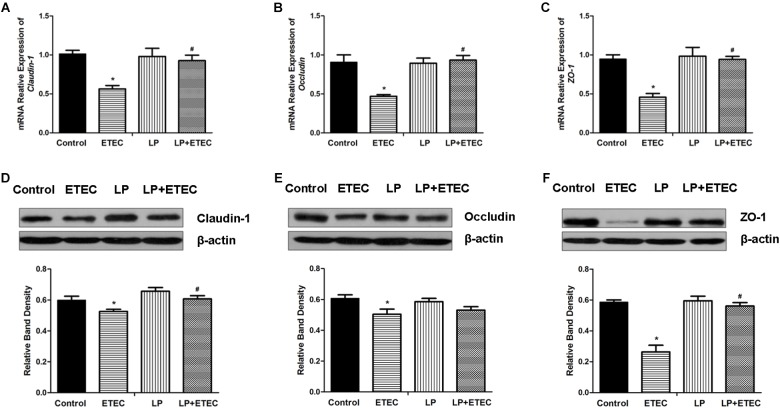
Relative mRNA transcript and protein levels of TJ proteins in IPEC-J2 cells left untreated or pretreated with *L. plantarum* ZLP001 (LP, 10^8^ CFU mL^−1^) for 6 h, and then challenged or not with 10^6^ CFU mL^−1^ ETEC for 3 h. mRNA levels of *claudin-1* **(A)**, *occludin* **(B)**, and *ZO-1* **(C)** were standardized to that of *GAPDH*. Expression levels relative to non-treated controls were calculated by the 2^−ΔΔC_T_^ method. TJ protein levels were assessed by immunoblotting. Data are western blotting results and gradation analysis of claudin-1 **(D)**, occludin **(E)**, ZO-1 **(F)**. Values are shown as the means ± SE of three independent experiments. ^∗^*P* < 0.05 vs. non-treated controls; ^#^*P* < 0.05 vs. ETEC alone.

Abundances of *claudin-1* (**Figure 2A**), *occludin* (**Figure 2B**), and *ZO-1* (**Figure 2C**) transcripts in IPEC-J2 cells after bacterial treatments were examined by RT-qPCR (**Figure 2**). After IPEC-J2 cells were incubated with ETEC alone, the mRNA expression levels of these genes were significantly decreased (*P* < 0.05) relative to those in the untreated control. *L. plantarum* ZLP001 treatment alone had no significant influence on TJ mRNA expression as compared to the untreated control. *L. plantarum* ZLP001 pretreatment significantly (*P* < 0.05) abrogated the decreases in TJ-related mRNA expression caused by ETEC infection.

Differences in TJ protein expression after the bacterial treatments were examined by western blotting. Expression levels of claudin-1 (**Figure 2D**), occludin (**Figure 2E**), and ZO-1 (**Figure 2F**) were significantly lower (*P* < 0.05) in cells exposed to ETEC than in the untreated controls. These results were consistent with those for mRNA expression. *L. plantarum* ZLP001 treatment alone did not significantly affect protein expression relative to the untreated control. *L. plantarum* ZLP001 pretreatment negated the reduction in claudin-1 (**Figure 2D**) and ZO-1 (**Figure 2F**) abundance caused by ETEC treatment.

### Effects of *L. plantarum* ZLP001 on the Epithelial Immunological Barrier

**FIGURE 3:**
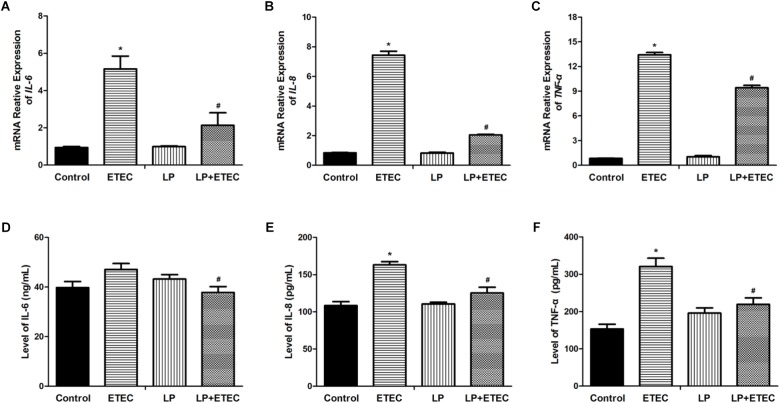
Relative mRNA transcript levels and concentrations of proinflammatory cytokines in the culture supernatant of IPEC-J2 cells left untreated or pretreated with *L. plantarum* ZLP001 (LP, 10^8^ CFU mL^−1^) for 6 h then either unchallenged or challenged with 10^6^ CFU mL^−1^ ETEC for 3 h. mRNA levels of *IL-6* **(A)**, *IL-8* **(B)**, and *TNF-α* **(C)** were standardized to that of *GAPDH*. Expression levels relative to non-treated controls were calculated by the 2^−ΔΔC_T_^ method. Protein expression of IL-6 **(D)**, IL-8 **(E)**, and TNF-α **(F)** was assessed by ELISA. Values are shown as the means ± SE of three independent experiments. ^∗^*P* < 0.05 vs. non-treated controls; ^#^*P* < 0.05 vs. ETEC alone.

Proinflammatory cytokines in IPEC-J2 cells were quantified after the treatments (**Figures 3A–C**). Incubation with ETEC alone significantly upregulated *IL-6*, *IL-8*, and *TNFα* transcripts. Treatment with *L. plantarum* ZLP001 alone had no significant effect on cytokine expression. However, pretreatment with *L. plantarum* ZLP001 prior to the ETEC challenge reduced cytokine expression in the IPEC-J2 cells to levels lower than those observed after ETEC treatment alone (*P* < 0.05). Therefore, *L. plantarum* ZLP001 reduced the ETEC-induced upregulation of proinflammatory cytokines.

ELISA was used to verify the protective effect of *L. plantarum* ZLP001 on epithelial immunological function at the protein level after ETEC challenge (**Figures 3D–F**). The results confirmed that, while ETEC did not significantly induced IL-6, *L. plantarum* ZLP001 pretreatment suppressed the increases in IL-6, IL-8, and TNFα secretion in IPEC-J2 cells challenged with ETEC relative to the levels observed in cells incubated with ETEC alone.

### Effects of *L. plantarum* ZLP001 on HDP Production

**FIGURE 4:**
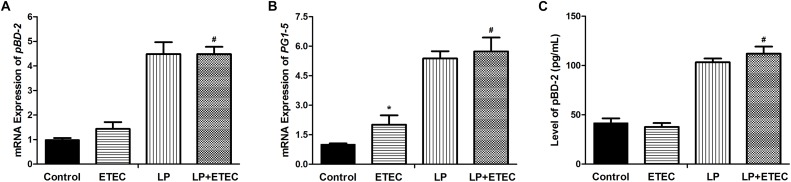
Relative mRNA transcript levels of *pBD2* **(A)** and *PG1*-*5* **(B)** and concentrations of pBD2 **(C)** in the culture supernatant of IPEC-J2 cells left untreated or pretreated with *L. plantarum* ZLP001 (LP, 10^8^ CFU mL^−1^) for 6 h and then challenged or not with 10^6^ CFU mL^−1^ ETEC for 3 h. mRNA expression levels were standardized to that of *GAPDH*. Expression levels relative to non-treated controls were calculated by the 2^−ΔΔC_T_^ method. The pBD2 concentration was assessed by ELISA. Values are shown as the means ± SE of three independent experiments. ^∗^*P* < 0.05 vs. non-treated controls; ^#^*P* < 0.05 vs. ETEC alone.

The modulatory effect of *L. plantarum* ZLP001 on the innate immune response was evaluated by measuring porcine HDP mRNA expression (**Figures 4A,B**). Cathelicidins and β-defensins are the two main mammalian HDP families. We selected *pBD2* (a β-defensin, **Figure 4A**) and *PG1-5* (a cathelicidin, **Figure 4B**) as target genes in this study. The results showed that treatment with *L. plantarum* ZLP001 significantly induced mRNA expression of both HDPs in IPEC-J2 cells (*P* < 0.05). ETEC exposure had no significant effect on *pBD2* expression, but significantly induced *PG1-5* expression. Challenge with ETEC 3 h after *L. plantarum* ZLP001 pretreatment had no significant effect on the HDP expression levels observed after incubation with *L. plantarum* ZLP001 alone.

Effects of bacterial treatment on pBD2 secretion were evaluated by ELISA (**Figure 4C**). The result was consistent with pBD2 mRNA expression. Exposure to *L. plantarum* ZLP001 significantly induced pBD2 secretion in IPEC-J2 cells. In contrast, ETEC treatment had no significant effect on pBD2 secretion. Challenge with ETEC 3 h after *L. plantarum* ZLP001 pretreatment had no significant influence on pBD2 secretion by *L. plantarum* ZLP001 treatment alone.

### Sequencing Results

Sequencing of the amplified 16S rRNA genes produced 373,846 reads after quality checks. An average of 37,385 ± 4,742 reads were obtained for each sample. Among the high-quality sequences, >99% were >400 bp. The average read length for each sample was 435 bp. Reads were clustered into 3,746 OTUs using a 97% similarity cut-off. We obtained 329–404 OTUs per sample. Based on rarefaction analysis, the sequencing depth adequately reflected species richness, suggested that the Illumina MiSeq sequencing system detected most of the fecal bacterial diversity in our study.

### Effects of *L. plantarum* ZLP001 on Alpha Diversity of Fecal Microbiota

We used the Chao1, ACE, Shannon, and Simpson indices to estimate fecal microbiome taxon abundance and diversity (**Table 1**). *L. plantarum* ZLP001-treated groups exhibited higher diversity than the control group according to the Shannon and Simpson indices. Nevertheless, the difference was not significant (*P* > 0.05). *L. plantarum* ZLP001 treatment had no significant effect on fecal microbiota abundance according to the Chao1 and ACE indices. Good’s coverage was >99.6% for all samples. Thus, the dominant bacterial phylotypes present in the feces were captured by this analysis.

**Table 1:** Effects of *L. plantarum* ZLP001 treatment on average richness and diversity of bacterial community in piglet feces.

Treatment	Abundance		Coverage	Diversity	
	ACE	Chao1		Shannon	Simpson
Control	559.3	569.8	0.9968	4.31	0.0394
*L. plantarum* ZLP001	556.5	565.7	0.9971	4.47	0.0313
SEM	14.15	16.21		0.730	0.00355
*P*-value	0.929	0.908		0.311	0.282

### Effects of *L. plantarum* ZLP001 on Fecal Microbiota Composition

**FIGURE 5:**
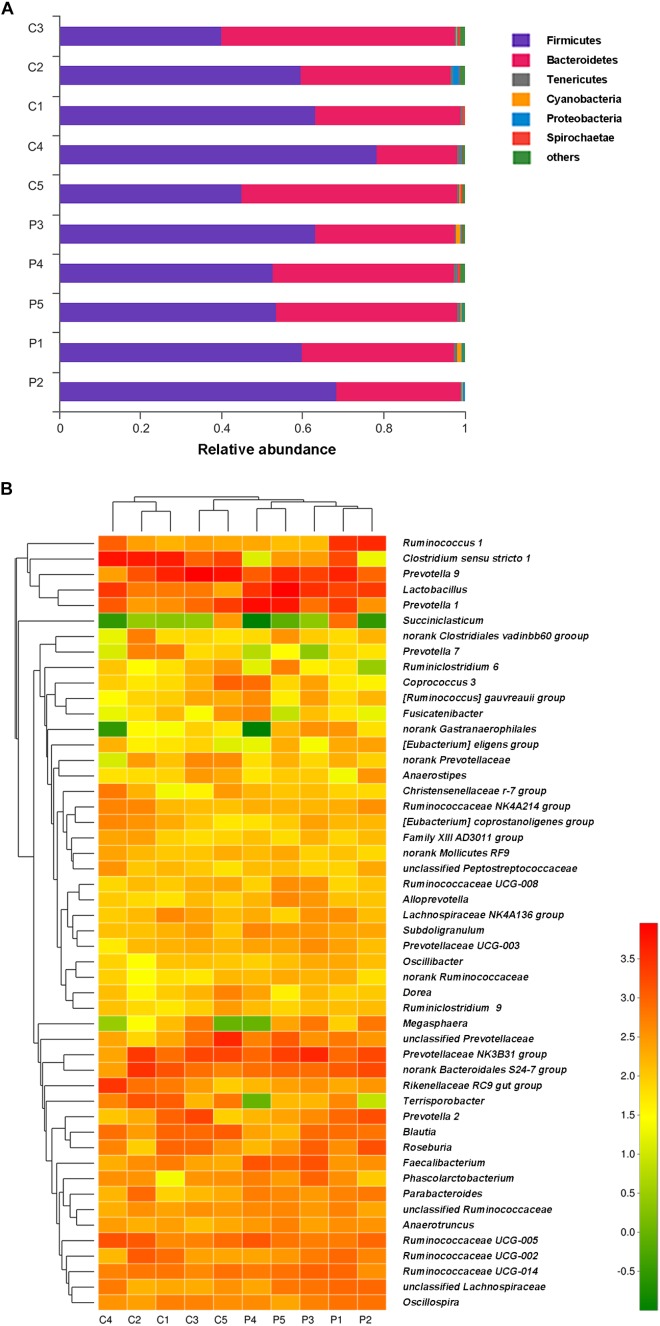
Phylum-level microbiota profile of *L. plantarum* ZLP001-treated piglets as compared to that of placebo-treated control piglets **(A)**. Stacked column chart showing the relative phylum-level bacterial abundance per fecal sample. Genus-level microbiota profile of *L. plantarum* ZLP001-treated piglets as compared to that of placebo-treated control piglets **(B)**. The heatmap shows genera whose relative abundance was >0.1%. Relative abundance is indicated by a color gradient from green to red, with green representing low abundance and red representing high abundance. C and P represent the control and the probiotic-treated group, respectively. Numbers represent individual animals.

**FIGURE 6:**
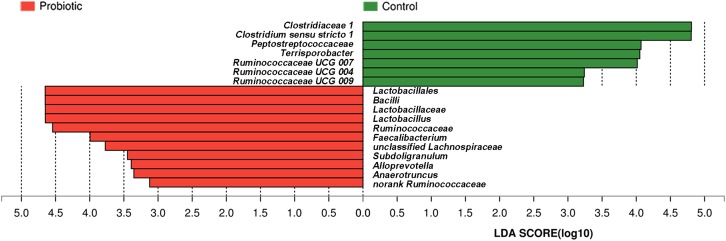
Differences between bacterial taxa in *L. plantarum* ZLP001-treated piglets and placebo-treated control piglets. The histogram shows differentially abundant bacteria in the treatment and control groups ranked by linear discriminant analysis (LDA) scores. Red bars (negative LDA scores) represent bacteria that are more abundant in probiotic-treated fecal samples than in controls. Green bars (positive LDA scores) represent bacteria that are more abundant in placebo-treated fecal samples than in probiotic-treated fecal samples.

Taxon-dependent analysis was used to compare microbiota compositions of the feces from piglets treated with *L. plantarum* ZLP001 and those receiving the placebo (**Figure 5**). Firmicutes and Bacteroidetes were the most abundant phyla in both groups and accounted for >97% of the total sequences on average. Firmicutes was the dominant phylum and constituted 58.1% in all treatments. Bacteroidetes accounted for 39.6%. Other phyla were present at lower frequencies. **Figure 5B** shows a hierarchically clustered heatmap of the fecal microbiota composition at the genus level. *Prevotella* was the most abundant; it accounted for an average of 21.5% of the sequences in all treatments by the end of the experiment. *Clostridium sensu stricto 1* (14.2%) was identified in the control piglets. *Lactobacillus* (12.8%) was detected in the *L. plantarum* ZLP001-treated piglets. LEfSe analysis indicated no significant differences between the placebo- and *L. plantarum*-treated piglets at the phylum level in terms of relative OTU abundance. Significant differences were observed between groups at several other taxa (**Figure 6**). The probiotic-treated group was enriched in Bacilli at the class level, Lactobacillales at the order level, Lactobacillaceae and Ruminococcaceae at the family level, and *Alloprevotella*, *Anaerotruncus*, *Faecalibacterium*, *Lactobacillus*, *Subdoligranulum*, unclassified *Lachnospiraceae*, and no-rank *Ruminococcaceae* at the genus level. However, it was depleted in Clostridiaceae_1 and Peptostreptococcaceae at the family level and *Clostridium sensu stricto 1*, *Terrisporobacter*, *Ruminococcaceae*_*UCG*_*007*, *Ruminococcaceae*_*UCG*_*004*, and *Ruminococcaceae*_*UCG*_*009* at the genus level. Fecal microbiota composition PCoA revealed that *L. plantarum* ZLP001 treatment significantly affected overall fecal microbiota composition. The microbiota communities in the piglets treated with *L. plantarum* ZLP001 were clustered together and were distinctly separated from those of the control pigs (**Supplementary Figure S1**).

### Effects of *L. plantarum* ZLP001 on Fecal SCFA Concentrations

**Table 2** shows the SCFA concentrations in piglet feces. *L. plantarum* ZLP001 treatment increased butyric acid concentrations relative to those in the controls (*P* = 0.068). Acetic and propionic acid concentrations did not significantly differ between the placebo and *L. plantarum* ZLP001 treatments (*P* > 0.05).

**Table 2:** Effects of *L. plantarum* ZLP001 on SCFA concentration (mmol kg^−1^) in piglet feces.

Short chain fatty acid	Acetic acid (AA)	Propionic acid (PA)	Butyric acid (BA)
Control	28.3	14.7	8.2
*L. plantarum* ZLP001	30.3	13.8	9.3
SEM	1.26	0.52	0.48
*P-value*	0.276	0.117	0.068

### Correlation Between SCFA Concentrations and Fecal Microbiota

**FIGURE 7:**
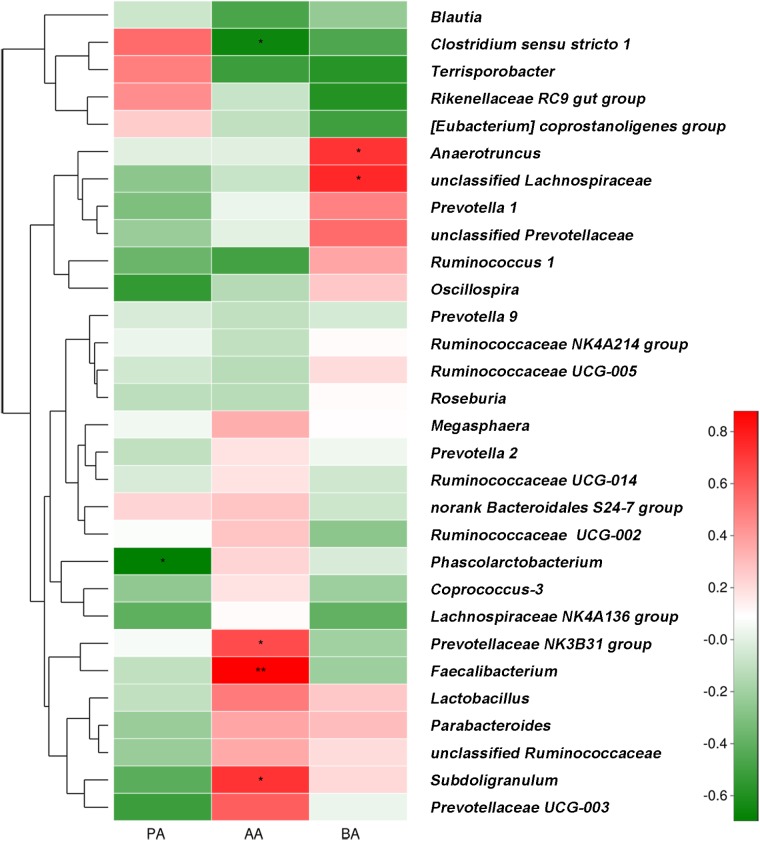
Correlations between relative generic abundance and SCFA concentrations in feces obtained from post-weaning piglets. Only the relative abundances of the top 30 bacterial genera are shown. Correlation is indicated by a color gradient from green to red based on Spearman’s correlation coefficients. Asterisks in red cells represent significant positive correlations (^∗^*P* < 0.05; ^∗∗^*P* < 0.01). Asterisks in green cells represent significant negative correlations (*P* < 0.05). AA, acetic acid; PA, propionic acid; BA, butyric acid.

Correlations between SCFA concentration and fecal bacterial abundance (relative abundance of the top 30 genera) are shown in **Figure 7**. The results demonstrated positive associations between butyric acid concentration and *Anaerotruncus* (*r* = 0.721, *P* = 0.019) and unclassified_f_*Lachnospiraceae* (*r* = 0.758, *P* = 0.011) abundance. Acetic acid concentration was positively correlated with *Faecalibacterium* (*r* = 0.879, *P* = 0.001), *Subdoligranulum* (*r* = 0.721, *P* = 0.019), and *Prevotellaceae*_NK3B31_group (*r* = 0.648, *P* = 0.043) abundance. It was negatively correlated with *Clostridium sensu stricto* *1* abundance (*r* = −0.661, *P* = 0.038). Propionic acid concentration was negatively correlated with *Phascolarctobacterium* abundance (*r* = −0.697, *P* = 0.025).

## Discussion

Using porcine IPEC-2 cells as a model, we demonstrated that *L. plantarum* ZLP001 plays multiple protective roles in epithelial barrier regulation. The present study showed that ETEC treatment significantly increased gut permeability to FD-4, whereas treatment with probiotic *L. plantarum* ZLP001 alone had no significant effect on gut permeability. These findings corroborate those of a previous study, in which probiotic *L. reuteri* did not significantly change FD-4 fluorescence intensity in IPEC-J1 cells (Wang et al., 2016). On the other hand, *L. plantarum* ZLP001 pretreatment significantly suppressed the increase in gut permeability caused by ETEC infection, suggesting that *L. plantarum* ZLP001 can alleviate epithelial damage caused by ETEC. This result was consistent with that of a previous study in which permeability to FD-4 indicated that *L. reuteri* treatment maintains the barrier integrity of IPEC-J1 cells exposed to ETEC (Wang et al., 2016).

TJ proteins play crucial roles in maintaining barrier integrity and function. They include transmembrane proteins such as claudins and occludins, and cytoplasmic scaffolding proteins, such as the ZO family, which have linking and sealing effects (Suzuki, 2013). In this study, relative TJ transcript and protein abundances were significantly reduced after ETEC infection. However, these reductions were abrogated by pretreatment with *L. plantarum* ZLP001. Previous studies using various probiotic strains reported similar results *in vivo* and in *vitro* (Yang et al., 2014; Wu et al., 2016). Therefore, probiotic *L. plantarum* ZLP001 may fortify intestinal epithelial resistance to pathogens by maintaining TJ protein abundance.

Cytokines play significant regulatory roles in the intestinal inflammatory response. Several studies have demonstrated the effects of probiotics on cytokine expression. Nevertheless, this regulatory action varies with strain. *L. reuteri* ACTT 6475 shows immunosuppressive action by inhibiting *TNFα* overexpression in LPS-activated human monocytoid THP1 cells (Lin et al., 2008). However, *L. reuteri* ACTT 55730 significantly stimulates TNF-α production as an immunostimulatory action (Jones and Versalovic, 2009). In the present study, *L. plantarum* ZLP001 *per se* did not influence the expression of proinflammatory cytokines, but inhibited their ETEC-induced overexpression, thus exerting an immunosuppressive action. Certain pro-inflammatory cytokines reportedly are associated with pathogen-induced TJ protein changes (Otte and Podolsky, 2004). ETEC K88 substantially increases IL-8 and disrupts the membrane barrier; however, this disruption can be alleviated by *L. plantarum* pretreatment (Wu et al., 2016). We obtained similar results in the present study. ETEC-induced increases in IL-6-, IL-8-, and TNF-α expression were effectively counteracted by *L. plantarum* ZLP001 pretreatment. This observation was consistent with the FD-4 assay results and TJ protein expression levels. Similar findings indicated that *L. reuteri* inhibits *TNF-α* expression and may protect TJ proteins (Yang et al., 2015). High-throughput sequencing analysis of post-weaning piglet feces after *L. plantarum* ZLP001 or control treatment revealed relatively low abundances of certain bacterial genera in the probiotic-treated group. Some of these are associated with various pathological conditions, e.g., *Peptostreptococcaceae incertae* *sedi*, which is dominant in viral diarrhea (Ma et al., 2011). Wang et al. (2017) reported that *IL-1β* and *TNF-α* transcript levels were positively correlated with *Clostridium sensu stricto 1* enrichment in the sheep colon. In the present study, *Clostridium sensu stricto 1* was significantly less abundant in the probiotic-treated than in the control group. Certain *Clostridium* spp. are harmful to host health. Epithelial inflammation observed in weaned piglets may be correlated with *Clostridium sensu stricto 1* enrichment in their intestinal mucosa (Wang et al., 2017). The protective effect of probiotics in terms of epithelial immunity may be partially explained by microbiota modulation. One limitation of the present study was that we did not evaluate proinflammatory cytokines in the piglet intestinal tissue and thus, we could not analyze the correlation with microbiota abundance. Such relationships merit further investigation.

The secretion of HDPs, which exert both antimicrobial and immunomodulatory activities, is an epithelial innate immunity mechanism (Bevins et al., 1999; Zhang et al., 2000). Enhancing endogenous HDP synthesis improves the early response to bacterial infection and inflammation (Veldhuizen et al., 2008). Nutrients, such as VD_3_, butyrate, and zinc induce HDP secretion (Talukder et al., 2011; Zeng et al., 2013; Merriman et al., 2015). Probiotics can also stimulate HDP expression (Schlee et al., 2008; Liu et al., 2017). In the present study, increased *pBD2* and *PG1-5* expression and pBD2 secretion were observed after *L. plantarum* ZLP001 treatment, suggesting that this strain can induce HDPs, to protect against bacterial infection. Similar results have been reported for the probiotic strain *L. reuteri* I5007 (Liu et al., 2017). The administration of synthetic HDPs reportedly can improve weaned piglet growth performance, nutrient digestion and assimilation, intestinal health, and antioxidant capacity (Xiao et al., 2013; Yoon et al., 2013; Yu et al., 2017). Therefore, the induction of HDP expression by *L. plantarum* ZLP001 may be correlated with the improved growth and reduced risk of diarrhea reported in our previous studies. SCFAs, especially butyrate, induce HDPs (Zeng et al., 2013). Butyrate production by enteric microbiota is the only microbial stimulus capable of inducing HDP expression (Schauber et al., 2003). Most butyrate-producing bacteria belong to *Clostridium* clusters IV and XIVa. Butyrate metabolism has been observed in species of *Faecalibacterium* and *Anaerotruncus*, e.g., *Faecalibacterium prausnitzii* and *Anaerotruncus colihominis* are butyrate producers (El Aidy et al., 2013; Wrzosek et al., 2013). *A. colihominis* has been shown to specifically colonize the lumen whereas *F. prausnitzii* is enriched in the mucus (Van den Abbeele et al., 2013). In the present study, *Faecalibacterium* spp. and *Anaerotruncus* spp. were significantly abundant in *L. plantarum* ZLP001-treated piglets. Therefore, increasing the abundances of these genera may elevate butyrate levels and epithelial HDP expression. The present study confirmed a positive correlation between fecal butyric acid and *Anaerotruncus* spp. abundance after *L. plantarum* ZLP001 treatment. In addition, *Faecalibacterium* spp. abundance showed a positive correlation with acetic acid concentration. Acetic acid can be converted to butyrate by *Eubacterium rectale* (Duncan and Flint, 2008), which may enhance HDP expression. The modulation of butyrate producers with probiotics to generate butyrate to stimulate HDP levels in the epithelium may thus be a meaningful approach for future interventions that aim to improve intestinal balance. Few studies have focused on the association between HDP production and probiotic function. Future studies involving probiotical and the non-pathogenic enteric bacterial regulation of antimicrobial peptides may elucidate the beneficial effects of probiotics against pathogen infection.

The piglet intestinal microbiota undergoes substantial dynamic changes after weaning, and this alteration can be associated with severe disorders and bowel disease. In the present study, fecal bacterial communities were dominated by Firmicutes and Bacteroidetes, regardless of treatment. This result was expected because the colon is a strictly anaerobic environment and most of the species within these phyla are anaerobic. Similar results were reported in previous pig studies (Konstantinov et al., 2004; Kim and Isaacson, 2015). *Prevotella*, which is associated with hemicelluloses degradation, reportedly is the predominant genus in piglets at nursery stage (Konstantinov et al., 2004). A high *Prevotella* spp. abundance may be essential for post-weaning piglets to be able to digest plant-based diets. Post-weaning increases in the proportions of *Lactobacillus* spp. are desirable and beneficial. Previous studies have shown that oral administration of lactic acid bacteria enhances the relative abundance of intestinal *Lactobacillus* spp. in weaned piglets (Hu et al., 2015; Zhang et al., 2016). In the present study, dietary *L. plantarum* ZLP001 supplementation significantly increased *Lactobacillus* spp. abundance in the post-weaning piglet intestine. *L. plantarum* ZLP001 may produce molecules that stimulate *Lactobacillus* spp. growth in the piglet intestine (Ohashi et al., 2001). Alternatively, the observed increase in *Lactobacillus* abundance may have resulted from the proliferation of the administered probiotic strain (Takahashi et al., 2007). Certain lactobacilli, such as *L. rhamnosus*, *L. reuteri*, and *L. plantarum*, protect TJ proteins after stress and infection and may, therefore, contribute to TJ integrity and intestinal barrier function (Yang et al., 2015; Blackwood et al., 2017). Further studies are needed to investigate specific species and their effects on piglet epithelial TJ proteins. The effects on gut microbiota composition and community observed after *L. plantarum* ZLP001 treatment in this study suggest that the modulation of the intestinal flora by *L. plantarum* ZLP001 helps to maintain a well-balanced gut microbiota, thereby improving the health and growth of pigs.

## Conclusion

**FIGURE 8:**
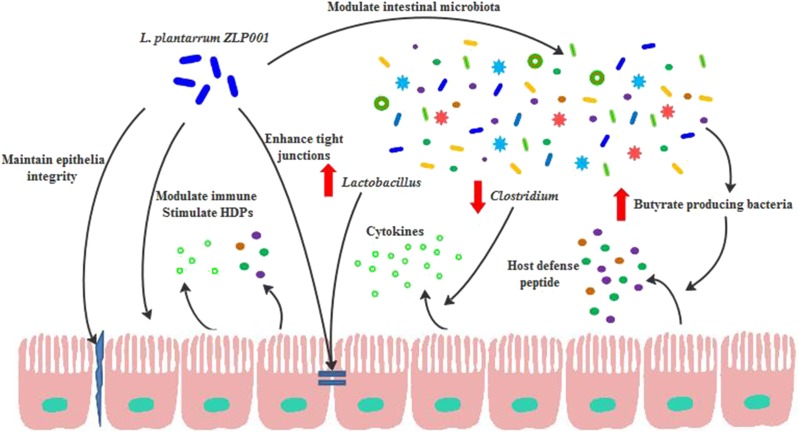
Suggested mechanism by which *L. plantarum ZLP001* protects intestinal barrier function from weaning stress. *L. plantarum ZLP001* functions not only through maintaining epithelial integrity, improving TJs, regulating the immune response, and stimulating HDPs, but also via modulating the intestinal indigenous microbiota. Modulated microbiota and alterations in certain bacterial taxa in turn enhance epithelial function.

Our IPEC-J2 model demonstrated that *L. plantarum* ZLP001 enhances intestinal barrier function by (1) maintaining epithelial integrity and preventing ETEC-induced gut permeability, (2) forming TJs and reducing ETEC-induced TJ damage, (3) modulating immune function and repressing the ETEC-induced immune response, and (4) inducing the secretion of antimicrobial peptides to protect against pathogens. Our study also indicated that *L. plantarum* ZLP001 supplementation improved gut bacterial ecology and barrier function in weaned piglets by (1) reducing the abundance of certain bacterial species correlated with proinflammatory cytokine expression, (2) modulating butyrate-producing enteric microbiota to induce epithelial HDP expression, and (3) enhancing intestinal *Lactobacillus* abundance to improve the gut microbiota composition and reinforce TJs (**Figure 8**). Elucidation of the mechanisms by which probiotics act on the intestinal barrier will promote their use in livestock production. In addition, further animal studies are required to determine how *L. plantarum* ZLP001 protects the piglet intestinal barrier both before and after pathogenesis.

## Author Contributions

JW and HJ conceived and designed the experiments. JW, SW, HL, and DZ performed the experiments. JW and WZ analyzed the data. YW contributed reagents and materials. JW and HJ helped to draft the manuscript. All authors read and approved the final manuscript.

## Conflict of Interest Statement

The authors declare that the research was conducted in the absence of any commercial or financial relationships that could be construed as a potential conflict of interest.

## Footnotes

1 http://www.arb-silva.de

## Supplementary Material

FIGURE S1 Effects of *L. plantarum* ZLP001 on microbial community structure in piglet feces based on principal coordinate analysis (PCoA). PCoA plot showing microbiota clustering in various treatments. Each dot represents an individual sample. Red and green indicate control and probiotic-treated samples, respectively. C and P represent control and probiotic-treated groups, respectively. Numbers represent individual animals.

TABLE S1 Primers used in this study.

TABLE S2 Antibodies used in this study.

TABLE S3 Ingredients and chemical composition of the basal diet.
