# Supplementary figures and images for: Probiotic Lactobacillus plantarum Promotes Intestinal Barrier Function by Strengthening the Epithelium and Modulating Gut Microbiota
Source: Front Microbiol. 2018 Aug 24;9:1953. doi: 10.3389/fmicb.2018.01953 (PMC6117384; doi:10.3389/fmicb.2018.01953)

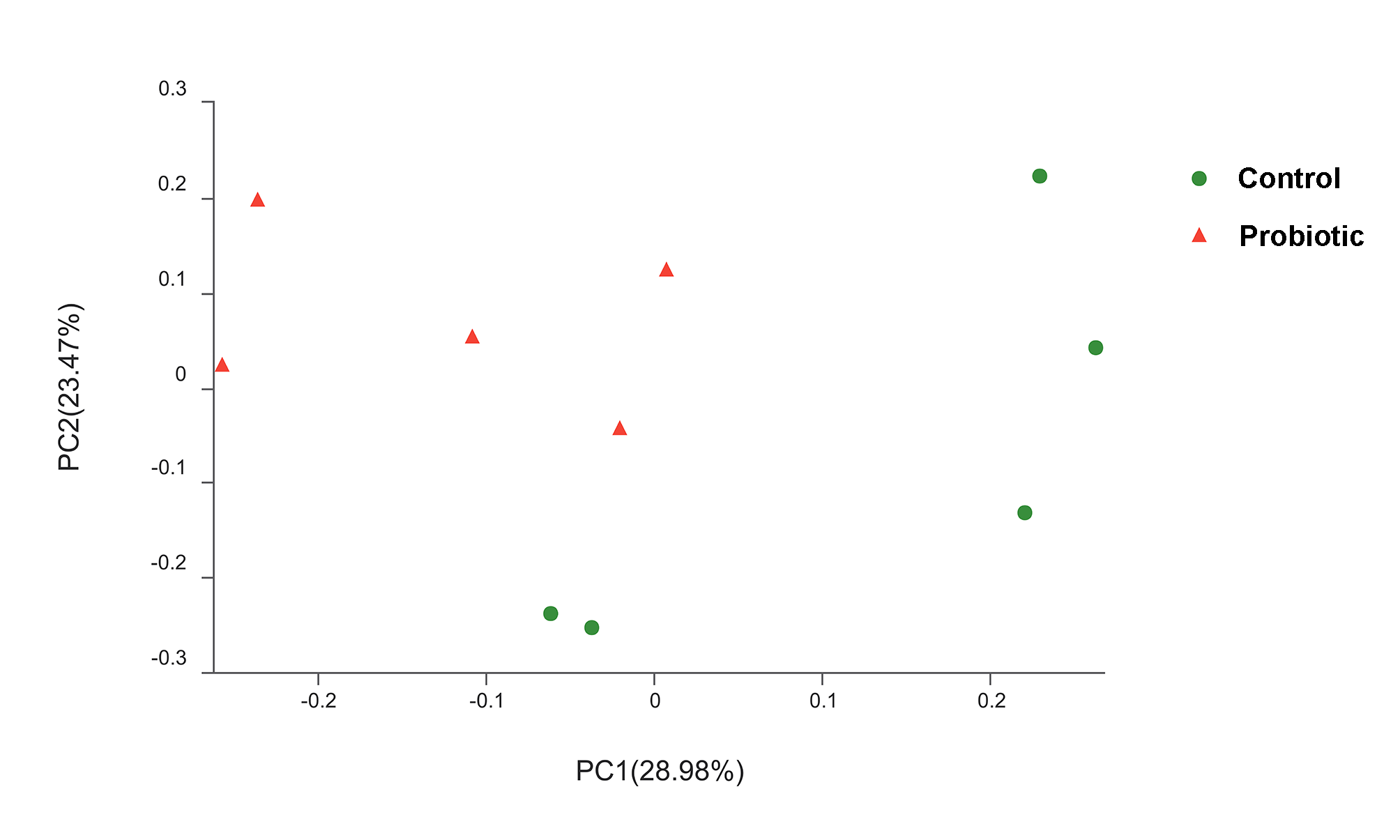

Supplement: FIGURE S1 — Effects of L. plantarum ZLP001 on microbial community structure in piglet feces based on principal coordinate analysis (PCoA). PCoA plot showing microbiota clustering in various treatments. Each dot represents an individual sample. Red and green indicate control and probiotic-treated samples, respectively. C and P represent control and probiotic-treated groups, respectively. Numbers represent individual animals. [file Image_1.tif]
