# Supplementary material for: Probiotic Lactobacillus plantarum Promotes Intestinal Barrier Function by Strengthening the Epithelium and Modulating Gut Microbiota
Source: Front Microbiol. 2018 Aug 24;9:1953. doi: 10.3389/fmicb.2018.01953 (PMC6117384; doi:10.3389/fmicb.2018.01953)
Supplement: TABLE S1 — Primers used in this study. [file Data_Sheet_1.docx]

**Supplementary Table S1.** Primers used in this study.

| Gene | Forward primer | Reverse primer | Product size (bp) |
| --- | --- | --- | --- |
| *GAPDH* | GCTACACTGAGGACCAGGTTG | CCTGTTGCTGTAGCCAAATTC | 146 |
| *Occludin* | TCGACTGGATAAAGAGCTGGA | TTACTTTTGTAATCCGCAGATCC | 114 |
| *Claudin* | TATCATCCTGGCCGTGCTA | CATCATCCACGCAGTTGGT | 71 |
| *ZO-1* | GAGTTTGATAGTGGCGTT | GTGGGAGGATGCTGTTGT | 298 |
| *IL6* | AAATGCTCTTCACCTCTC | TCACACTTCTCATACTTCTC | 106 |
| *IL8* | TTCGATGCCAGTGCATAAATA | CTGTACACCTTCTGCACCCA | 176 |
| *TNFα* | CCCCTCTGAAAAAGACACCA | TCGAAGTGCAGTAGGCAGAA | 180 |
| *pBD-2* | TGTCTGCCTCCTCTCTTCC | AACAGGTCCCTTCAATCCTG | 149 |
| *pBD-3* | CCTTCTCTTTGCCTTGCTCTT | GCCACTCACAGAACAGCTACC | 163 |
| *PG1-5* | ACGGTGAAGGAGACTGTG | CGCAGAACCTACGCCTACAA | 196 |

**Supplementary Table S2.** Antibodies used in this study.

| Antibody | Supplier | Dilution |
| --- | --- | --- |
| Rabbit polyclonal anti-claudin-1 | Santa Cruz Biotechnology, Dallas, TX, USA | 1:1,000 |
| Rabbit polyclonal anti-occludin | Abcam, Cambridge, UK | 1:1,000 |
| Rabbit polyclonal anti-ZO-1 | Immunoway, Plano, TX, USA | 1:1,000 |
| Mouse monoclonal anti-β-actin | Immunoway, Plano, TX, USA | 1:5,000 |

**Supplementary Table S3.** Ingredients and chemical composition of the basal diet.

| **Ingredient** | **Content (g kg^–1^)** |
| --- | --- |
| Corn | 600 |
| Soybean meal | 230 |
| Wheat bran | 50 |
| Fish meal | 20 |
| Whey | 50 |
| Soybean oil | 10 |
| Premix^a)^ | 40 |
| Chemical compositions | |
| Digestible energy^b)^ (MJ kg^-1^) | 13.8 |
| Crude protein^c)^ | 184.2 |
| Lysine^c)^ | 11.6 |
| Methionine^c)^ | 3.8 |
| Calcium^c)^ | 8.2 |
| Total phosphorus^c)^ | 6.5 |
| ^a)^ Provided per kg of complete diet: vitamin A, 11,000 IU; vitamin D_3_, 2,800 IU; vitamin E, 36 mg; niacin, 30 mg; pantothenic acid, 12.5 mg; riboflavin, 6.5 mg; menadione, 2.2 mg; vitamin B_12_, 0.045 mg; Fe, 150 mg; Zn, 120 mg; Cu, 100 mg; Mn, 35 mg; I, 0. 5 mg; Se, 0.3 mg.  ^b)^ Calculated nutrient levels.  ^c)^ Measured nutrient levels. | |
